# P-1158. Molecular Characterization of Extraintestinal Pathogenic Escherichia coli (ExPEC) from the Emerging Infections Program (EIP)─United States, 2023

**DOI:** 10.1093/ofid/ofaf695.1351

**Published:** 2026-01-11

**Authors:** Alyssa Kent, Heather N Grome, Joshua Brandenburg, Latasha Curtis, Ruben Raymond, Uzma Ansari, Alice Guh, Amy Gargis, Christopher Elkins, Susannah McKay

**Affiliations:** CDC, Atlanta, Georgia; Centers for Disease Control and Prevention, Atlanta, Georgia; Centers for Disease Control and Prevention, Atlanta, Georgia; Chenega Corporation, Atlanta, Georgia; Tanaq Support Services, Atlanta, Georgia; Centers for Disease Control and Prevention, Atlanta, Georgia; Centers for Disease Control and Prevention, Atlanta, Georgia; Centers for Disease Control & Prevnetion, Atlanta, GA; CDC, Atlanta, Georgia; CDC, Atlanta, Georgia

## Abstract

**Background:**

Extraintestinal pathogenic *E. coli* (ExPEC) is a major contributor to healthcare-associated infections (HAIs) and a leading cause of urinary tract infections (UTIs) and sepsis in the United States. Despite its substantial burden, comprehensive molecular epidemiological data on ExPEC in the United States remain limited.

**Methods:**

In 2023, the Emerging Infections Program piloted active laboratory- and population-based surveillance for invasive ExPEC (iEC) across nine U.S. sites (CA, CO, GA, MD, MN, NM, NY, OR, TN) from June to August and for urinary ExPEC (uEC) at three sites (CA, GA, NY) in August. Cases were defined as the first isolation of *E. coli* from a sterile site (iEC) or urine (uEC) from a surveillance-area resident in a 30-day period. All iEC and a random sample of uEC isolates were sent to the CDC for molecular subtyping via whole-genome sequencing (WGS) analysis including multi-locus sequence typing, *in silico* serotyping, and antibiotic resistance gene profiling.

**Results:**

The collection included 846 iEC and 939 uEC isolates. The sequence type (ST) 131 pandemic clone was the most prevalent lineage, comprising 19.0% and 15.4% of iEC and uEC isolates, respectively. *In silico* serotyping identified O25b as the dominant serotype, representing 16.2% and 12.2% of iEC and uEC isolates, respectively. All isolates harbored at least one beta-lactamase gene, three isolates harbored a carbapenemase gene (KPC-3 and OXA-244), and 11% harbored at least one extended-spectrum beta-lactamase gene. Additionally, approximately one-third of isolates harbored genetic determinants associated with multidrug resistance (defined as resistance to ≥3 antimicrobial classes).

**Conclusion:**

These findings enhance our understanding of ExPEC molecular epidemiology, highlighting the dominance of ST131 and O25b, along with widespread beta-lactamase and multidrug resistance. These baseline data are crucial considerations for vaccine development and antimicrobial stewardship to mitigate ExPEC’s public health impact.

**Disclosures:**

All Authors: No reported disclosures

